# Distraction behaviors of pedestrians in the city of Tabriz 

**Published:** 2019-08

**Authors:** Morteza Haghighi, Homayon Sadeghi-Bazargan, Fatemeh Bakhtari Aghdam, Mohammad Saadati

**Affiliations:** ^*a*^Road Traffic Injury Research Center, Health Management and Safety Promotion Research Institute, Tabriz University of Medical Sciences, Tabriz, Iran.

## Abstract

**Background:**

Traffic accidents represent the greatest economic burden across the world. Pedestrian behaviors ‎are very important due to the higher risk of road injuries among them, the purpose of this study ‎was to investigate of the Traffic Safety behavior of Pedestrians in Tabriz city.‎

**Methods:**

This study was a cross-sectional study to data were collected by observation and checklist. In ‎this study, the statistical population was all pedestrians in Tabriz 2nd district to 557 pedestrian ‎through census method and filming on a daily basis for six days and certain hours for 15 ‎minutes of filming from any intersections were studied. After filming, the information was ‎extracted from the checklist and analyzed using soft spss22. ‎

**Results:**

In this study, most people were in the age group of approximately 18-34 years (61.6%) And ‎male pedestrians were more than women (55.1%). According to the findings of this study, 24.3 ‎percent of pedestrians crossed the street, regardless of traffic signs and 20.6% when crossing the ‎intersection and the street had at least one type of distraction (mobile, hand-held, etc.) The most ‎common cause of distraction was talking with a friend (11.3%) and then using mobile (3.6%). ‎And overall 13.5 percent of pedestrians had unsafe traffic behavior during the crossing of the ‎street that 8.1% of them were accompanied by a type of distraction. Also, according to the ‎findings of this study, there was a significant relationship between distraction and unsafe ‎behavior and age group with pedestrian distraction (p <.05). ‎

**Conclusions:**

By identifying the factors associated with unsafe traffic behaviors and the role of distraction ‎factors in the unsafe behaviors of pedestrians, Behavioral and educational interventions should ‎be used to promote safe pedestrian behavior and to change the pedestrian -related rules.‎

**Keywords:**

Pedestrians, Distraction behaviors, Tabriz

